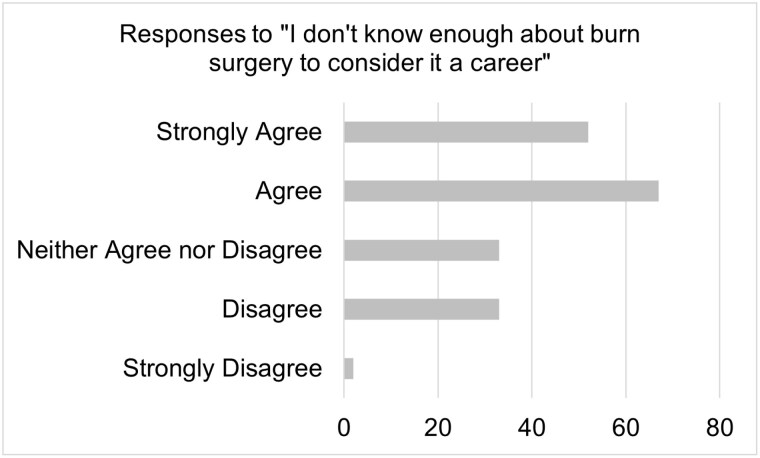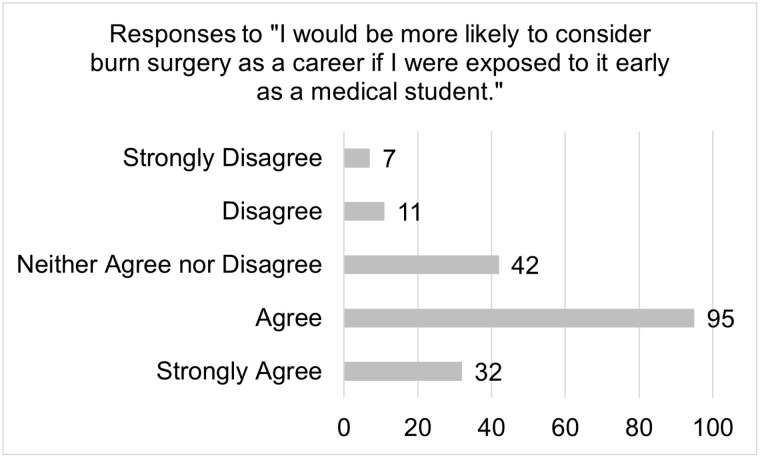# 618 Student Awareness of Burn Surgery as a Subspecialty

**DOI:** 10.1093/jbcr/iraf019.247

**Published:** 2025-04-01

**Authors:** Lauren Ellis, Duncan Nickerson, Taylor Cusick

**Affiliations:** University of Kansas School of Medicine; Kansas University Medical Center; University of Kansas School of Medicine

## Abstract

**Introduction:**

In 2004, a shortage of burn surgeons was cited in the literature, motivating the burn surgery community to improve recruitment and retention of burn surgeons. With this objective in mind, this study looked to analyze the awareness of burn surgery amongst medical students at our institution to determine how their understanding of burn surgery compares to other student populations so that informational resources can be tailored to fill the gaps in knowledge.

**Methods:**

An anonymous survey was sent to first- through fourth-year medical students at an institution affiliated with an ABA Certified Burn Center which allowed respondents to indicate their level of knowledge and interest in burn surgery using a 5-point Likert scale.

**Results:**

A total of 188 responses were collected and revealed that 64% of students do not know enough about burn surgery to consider it a career. This lack of knowledge is present throughout each class of medical students and includes not understanding the daily activities of a burn surgeon. Notably, 77% of students agree that they would attend burn surgery educational events; only 4.5% of students in their final year of school agreed to this statement. A majority of first- and second-year medical students, 66%, agreed that they would participate in shadowing of burn surgeons. Positive results indicate that 69% would be more likely to choose burn surgery with the help of a mentor and 68% agree they would be more likely to choose burn surgery if exposed to it earlier.

**Conclusions:**

Analysis confirmed that students at this institution have insufficient knowledge about burn surgery as a career. There is not a difference in this level of knowledge based on year in school, suggesting that students in the later years of their education have not had enough exposure to burn surgery, suggesting a lack of clinical exposure. These findings, coupled with the lack of interest in education events demonstrated by fourth year medical students and the affirmative interest of first- and second-year students, support our hypothesis that earlier exposure to burn surgery will be most beneficial. The positive response regarding interest in learning more about burn surgery provides evidence to move forward with subsequent research.

**Applicability of Research to Practice:**

To expand the power of these findings, the survey used in this study will be disseminated to students from another institution. It is hypothesized that this added population of medical students will report similar responses to the survey questions. Collecting more data will add support for the goal of creating online educational resources available to students that will aim to increase knowledge and awareness of the field of burn surgery, thus helping to strengthen the burn surgery workforce for the future.

**Funding for the Study:**

N/A